# Case Report: My lung broke my heart! Takotsubo cardiomyopathy due to pneumonia

**DOI:** 10.12688/f1000research.14546.1

**Published:** 2018-04-30

**Authors:** Navid Ahmed, Himali Gandhi, Daniel B. Sims

**Affiliations:** 1 Department of Medicine, Montefiore Medical Center, Albert Einstein College of Medicine, New York, New York, 10467, USA; 2 Division of Cardiology, Montefiore Medical Center, Albert Einstein College of Medicine, New York, New York, 10467, USA

**Keywords:** Takotsubo cardiomyopathy, Heart failure, Sepsis, Geriatrics, Cardiology, Pneumonia

## Abstract

Takotsubo cardiomyopathy (TTC), also known as stress-induced cardiomyopathy, is a cardiac syndrome that often mimics acute myocardial infarction. TTC is commonly triggered by physical or emotional stress; however, acute infection is a rarer etiology. This report concerns the case of an 82-year-old female who presented with non-positional and non-pleuritic chest pain, with an associated fever and cough and chest x-ray findings consistent with pneumonia. Cardiac enzymes and ECG findings were consistent with acute coronary syndrome (ACS); however, during coronary angiography, no coronary artery disease could explain the patient’s ACS. A post-catheterization echocardiogram revealed an ejection fraction of 25%, with apical akinesis. A repeat echocardiogram 4 weeks after presentation showed a normal EF and normal wall motion, confirming a diagnosis of TTC.

## Introduction

Takotsubo cardiomyopathy (TTC) is an etiology of chest pain that often mimics acute myocardial infarction. However, TTC presents with transient systolic dysfunction, which normalizes over time. Patients who typically present with TTC have an inciting physical or emotional stress event that is pinpointed as the etiology ^2^.

TTC is typically not associated with an infectious etiology as the inciting stressor; however, it has been rarely reported in previous case reports ^1^. Acute infection should be increasingly recognized as a possible trigger of TTC in a patient with chest pain.

## Case presentation

An 82-year-old female from a nursing home, with a history of dementia, hypertension, hyperlipidemia and coronary artery disease (CAD), and a drug-eluting stent placed in the left circumflex artery, presented from nursing home with recurrent non-positional and non-pleuritic chest pain, along with associated fever and cough. Further medical history could not be obtained from the patient owing to underlying dementia. Of note, the patient had a normal echocardiogram 10 days prior to presentation, with an ejection fraction (EF) of 60%. On presentation, her blood pressure was 113/78 mm Hg, and she had a pulse of 137 beats/min and a temperature of 101°F (38.3°C).

**Figure 1.**
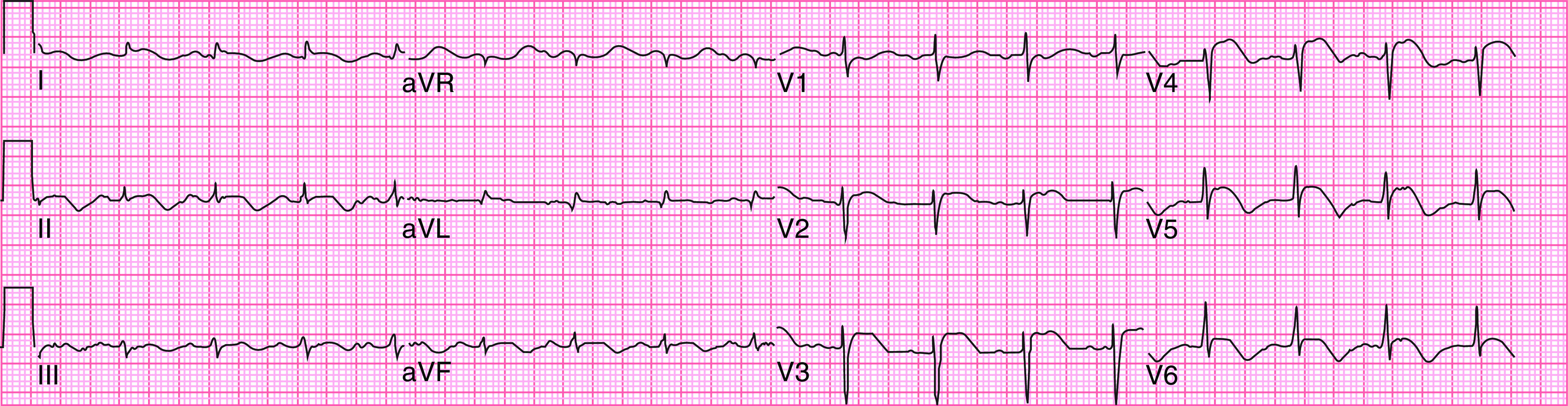
Electrocardiogram showing ST elevations in I, avL and V3–V6.

An electrocardiogram (ECG) showed ST elevations in I, avL, and V3–V6 ( Figure 1). A chest x-ray revealed pneumonia in the right middle and right lower lobes. The patient’s initial creatinine phosphokinase and troponin T were elevated at 330 U/l and 0.86 ng/ml, respectively, and ultimately peaked 6 h after presentation at 470 U/l and 1.39 ng/mll, respectively (normal creatinine phosphokinase < 200 U/l, normal Troponin T < 0.10 ng/ml.)

**Figure 2.**
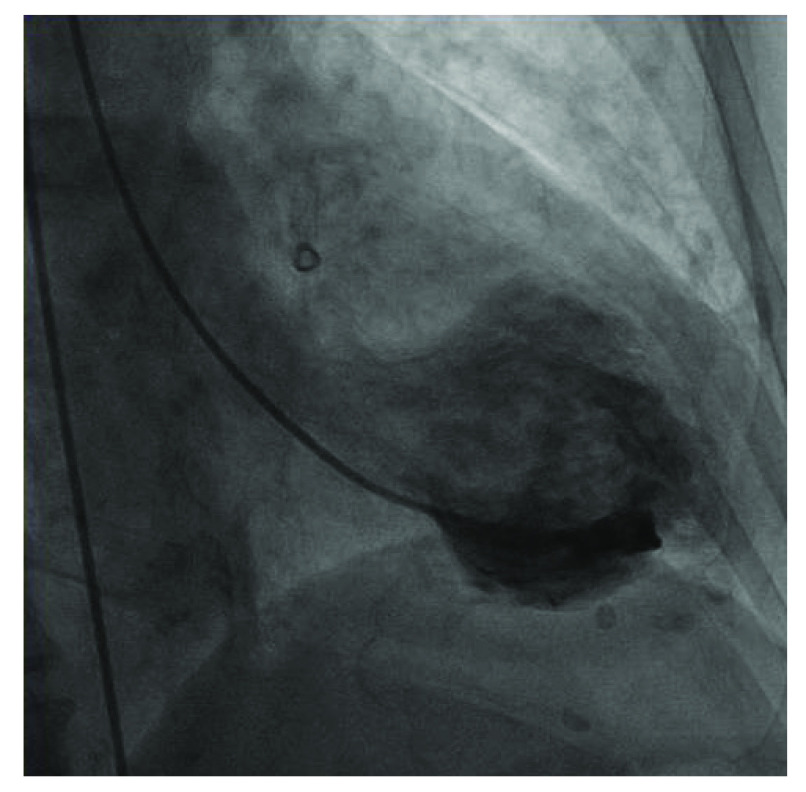
Left ventriculogram with a hypercontractile base and apical akinesis, typical in TTC.

The patient was taken for an emergency cardiac catheterization after verification of goals of care. Left ventriculogram showed a hypercontractile base and apical akinesis, with an EF of 20% ( Figure 2). There was no CAD, which explained the patient’s ECG or wall motion abnormalities, so no revascularization was performed.

The patient was placed on vancomycin (1 g every 12 h) and piperacillin–tazobactam (0.375 g every 6 h) for the treatment of pneumonia for a 7-day course, with an improvement in symptoms observed. She was continued on daily 81 mg aspirin, 50 mg metoprolol and 40 mg simvastatin, and started on 5 mg lisinopril. A repeat echocardiogram 4 weeks later revealed a normal EF and normal wall motion, confirming a diagnosis of TTC.

## Discussion

TTC, also known as stress-induced cardiomyopathy, is a cardiac syndrome that often mimics acute myocardial infarction and presents with transient systolic dysfunction of the apical segment of the left ventricle (LV), without the presence of obstructive coronary artery disease ^1^. The syndrome has a higher incidence in women than men ^1^.

Patients typically present with chest pain and dyspnea; however, cases have been reported of syncope, palpitations, hypotension and shock as the initial manifestation of TTC. Typically, TTC is preceded by a stressful event, such as tragic personal news, assaults, arguments or accidents ^2^. However, acute infection has been described as an uncommon etiology ^1, 3– 5^. Typical ECG findings include ST segment elevations and T-wave inversions. Coronary angiography typically does not reveal a culprit lesion and LV angiography shows LV apical ballooning.

The pathophysiological basis of TTC is still unknown, although potential mechanisms include multi-vessel coronary vasospasm, coronary microvascular dysfunction and catecholamine cardiotoxicity ^1, 2, 6^. TTC is commonly triggered by physical or emotional stress; however, rare cases of stress cardiomyopathy from acute infection have been reported ^1– 3^. A systematic review of sepsis and TTC hypothesized that inflammatory markers, such as tumor necrosis factor-α and interleukin-1β, along with other cytokines, act as a trigger for cardiac sympathetic nerve discharge, leading to an elevated norepinephrine state and then myocardial dysfunction ^2^. Another possible mechanism of TTC is myocardial ischemia due to inadequate coronary blood flow during sepsis ^3^. The patient in the present report presented with a baseline altered mental status secondary to dementia; no trigger for her episode of TTC other than her infection could be found.

TTC is a reversible cardiomyopathy that is typically associated with emotional stress; however, other inciting factors can trigger TTC. Infection is an uncommon inciting event for TTC. In patients who present with signs and symptoms of acute infection and chest pain, TTC should be considered in the differential diagnosis during the evaluation and workup of the patient.

## Consent

Written informed consent for publication of clinical details and images was obtained from a relative of the patient owing to the underlying dementia of the patient.
