# Supplementary material for: Case Report: My lung broke my heart! Takotsubo cardiomyopathy due to pneumonia
Source: F1000Res. 2018 Apr 30;7:518. [Version 1] doi: 10.12688/f1000research.14546.1 (PMC9568678; doi:10.12688/f1000research.14546.1)
Supplement: Peer review history

# Peer review: Reviewer response for version 1

**DOI:** 10.5256/f1000research.15833.r150419

**Published:** 2022-10-14

**Authors:** Mohammad Ullah Firoze

**Affiliations:** 1 Dhaka Medical College, Dhaka, Bangladesh

This is a well-written and described case report.

 TCM in during severe infectious disease is not uncommon. This was reported some authors. Overall, this is a good case report to raise awareness about TCM during severe infectious diseases also.

 There are two well-recognized diagnostic criteria for TCM. Mayo Clinic criteria and InterTAK criteria could have been discussed.

 Echocardiography before cardiac catheterization can give a good clue for diagnosis.

 There are some ECG features, like lack of ST depression is suggestive of TCM in relation to ACS.

 Myocarditis and pheochromocytoma are two important differential diagnoses, this could be mentioned. Specially myocarditis should be excluded when there is an associated infectious disease like a viral disease. Overall, this is a good case report to raise awareness about TCM during severe infectious diseases also.

Are enough details provided of any physical examination and diagnostic tests, treatment given and outcomes?

Partly

Is the case presented with sufficient detail to be useful for other practitioners?

Partly

Is sufficient discussion included of the importance of the findings and their relevance to future understanding of disease processes, diagnosis or treatment?

Partly

Is the background of the case’s history and progression described in sufficient detail?

Yes

Reviewer Expertise:

Clinical cardiology, echocardiography, Interventional cardiology.

I confirm that I have read this submission and believe that I have an appropriate level of expertise to confirm that it is of an acceptable scientific standard, however I have significant reservations, as outlined above.

# Peer review: Reviewer response for version 1

**DOI:** 10.5256/f1000research.15833.r147688

**Published:** 2022-10-06

**Authors:** Nicola Marziliano

**Affiliations:** 1 Università degli Studi del Molise, Campobasso, Italy

Taken by itself, pneumonia does not affect directly the heart, but it is a lung infection caused by either bacteria (i.e *. Klebsiella pneumoniae, Haemophilus influenzae, Moraxella catarrhalis, Pseudomonas aeruginosa,* etc ) or viruses (SARS-CoV-2, RSV, HxNx, etc) or even fungi. However, heart disease complications like congestive heart failure can cause a condition similar to pneumonia. Additionally, pneumonia tends to affect individuals who are also at high cardiovascular risk.

 Although acute cardiac events have been recognised as important complications in patients with pneumonia since the early 20th century, the magnitude of this problem has only recently begun to be appreciated fully. Under this light, the present work can add a further step in the disease (Takotsubo cardiomyopathy, TTC) understanding.

 Although, in the case presentation it is missing the link to the pneumonia symptoms and also the investigation of the underlying pneumonia’s causes (bacteria, viruses, fungi etc). I think the paper could be accepted after integrating shortly the clinical signs of the patient.

Are enough details provided of any physical examination and diagnostic tests, treatment given and outcomes?

Partly

Is the case presented with sufficient detail to be useful for other practitioners?

Partly

Is sufficient discussion included of the importance of the findings and their relevance to future understanding of disease processes, diagnosis or treatment?

Partly

Is the background of the case’s history and progression described in sufficient detail?

Partly

Reviewer Expertise:

Microbiology; Cardiomiopathies; Genetics; Inherited cardiovascular disorders; Channelopathies

I confirm that I have read this submission and believe that I have an appropriate level of expertise to confirm that it is of an acceptable scientific standard, however I have significant reservations, as outlined above.

# Peer review: Reviewer response for version 1

**DOI:** 10.5256/f1000research.15833.r58312

**Published:** 2020-01-27

**Authors:** Kunal Bhatt

**Affiliations:** 1 Department of Medicine, Division of Cardiology, School of Medicine, Emory University, Atlanta, GA, USA

Ahmed *et al.* report a concise case of an 82 year old female who developed Takotsubo cardiomyopathy (TTC) after an acute infectious bacterial Pneumonia.

 While TTC is usually described in relation to an emotional or physical stressor, this case is unique and contributes to the small existing literature of an infectious precipitant to the syndrome.

 The clear objective data of an existing echo 10 days prior to admission showing a normal LVEF adds to the clear development of this patient's syndrome as being induced by her multi-lobar pneumonia.

 This case report is written well with a clear focus on the objective of describing a unique case of TTC precipitated by multi-lobar Pneumonia. As sepsis and Pneumonia are fairly common diagnosis, this case report adds to the growing body of literature that describes infections as being another possible etiology of TTC in addition to emotinal and physical stressors.

Are enough details provided of any physical examination and diagnostic tests, treatment given and outcomes?

Yes

Is the case presented with sufficient detail to be useful for other practitioners?

Yes

Is sufficient discussion included of the importance of the findings and their relevance to future understanding of disease processes, diagnosis or treatment?

Yes

Is the background of the case’s history and progression described in sufficient detail?

Yes

Reviewer Expertise:

Heart failure, transplant cardiology, general cardiology, TTE, Amyloid cardiomyopathy

I confirm that I have read this submission and believe that I have an appropriate level of expertise to confirm that it is of an acceptable scientific standard.
